# Supplementary figures and images for: Population-wise labeling of sulcal graphs using multi-graph matching
Source: PLoS One. 2023 Nov 9;18(11):e0293886. doi: 10.1371/journal.pone.0293886 (PMC10635518; doi:10.1371/journal.pone.0293886)

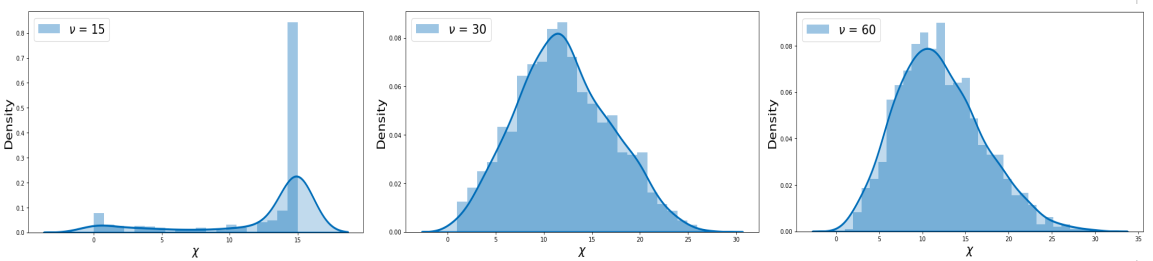

Supplement: S1 Fig — Effect on β-binomial mass function for different values of ν fixing α = 7.15 and β = 28.62. (PNG) [file pone.0293886.s001.png]

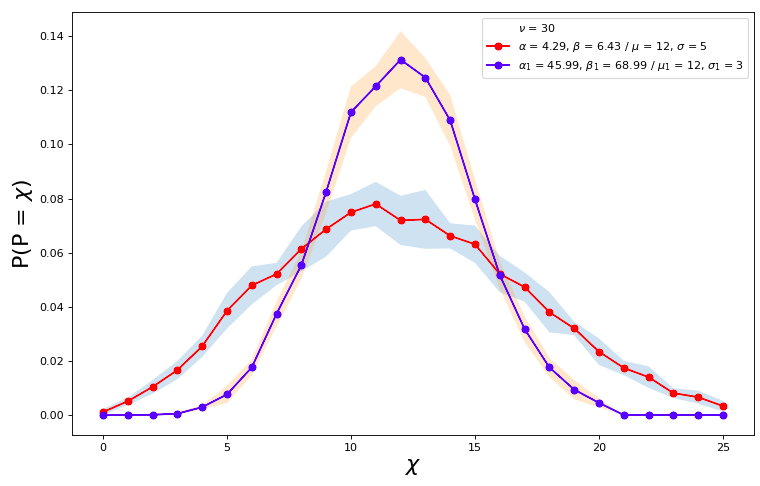

Supplement: S2 Fig — β-binomial distributions for identical mean: μ, μ1 = 12 but different standard deviations: σ = 3, σ1 = 5. The dotted lines signifies the mean of the distribution where as the shaded area is the standard deviation across 5 trials. (PNG) [file pone.0293886.s002.png]

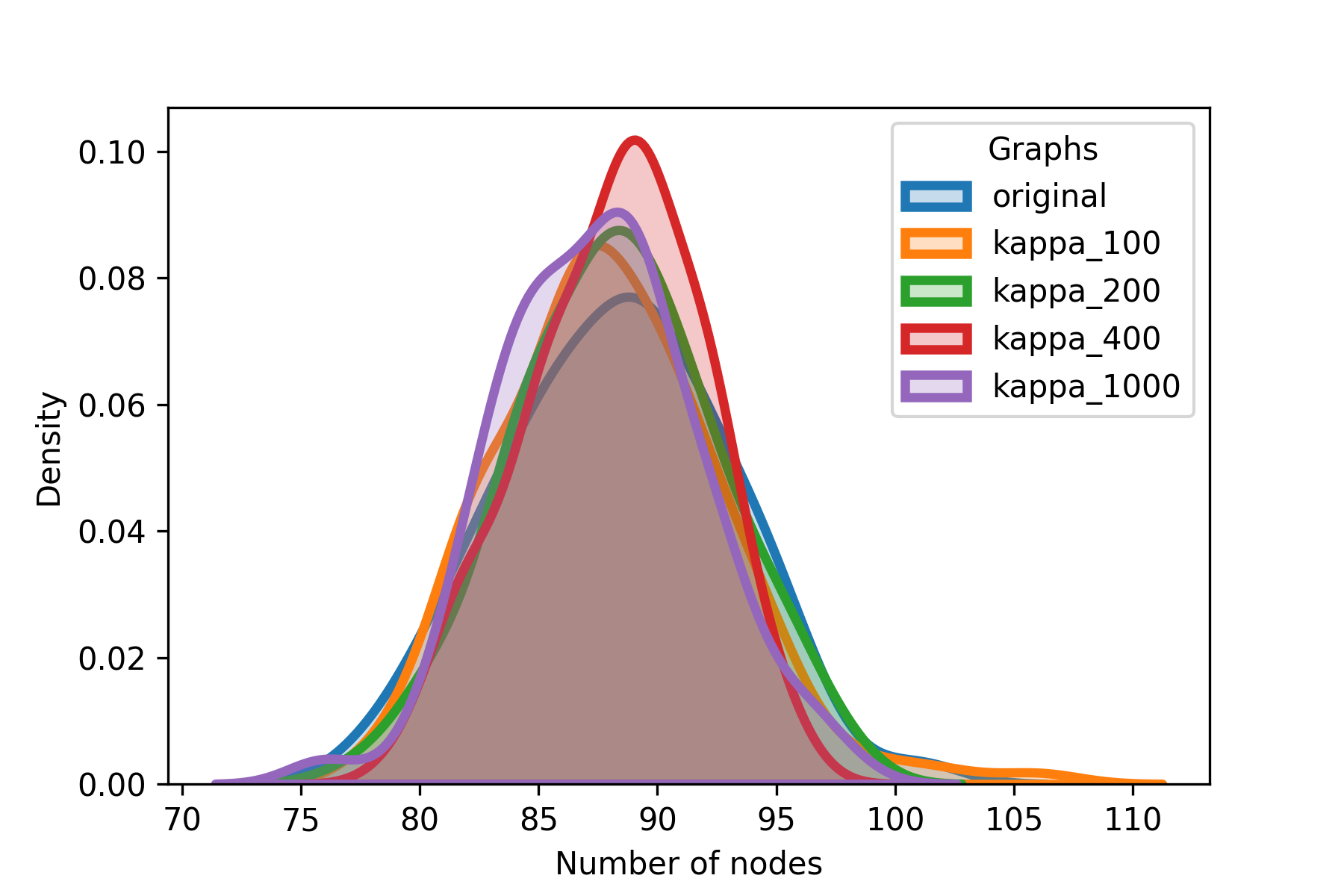

Supplement: S3 Fig — Distribution for number of node in the simulated population corresponding to different κ value with the distribution for number of nodes in the real sulcal graphs. (PNG) [file pone.0293886.s003.png]

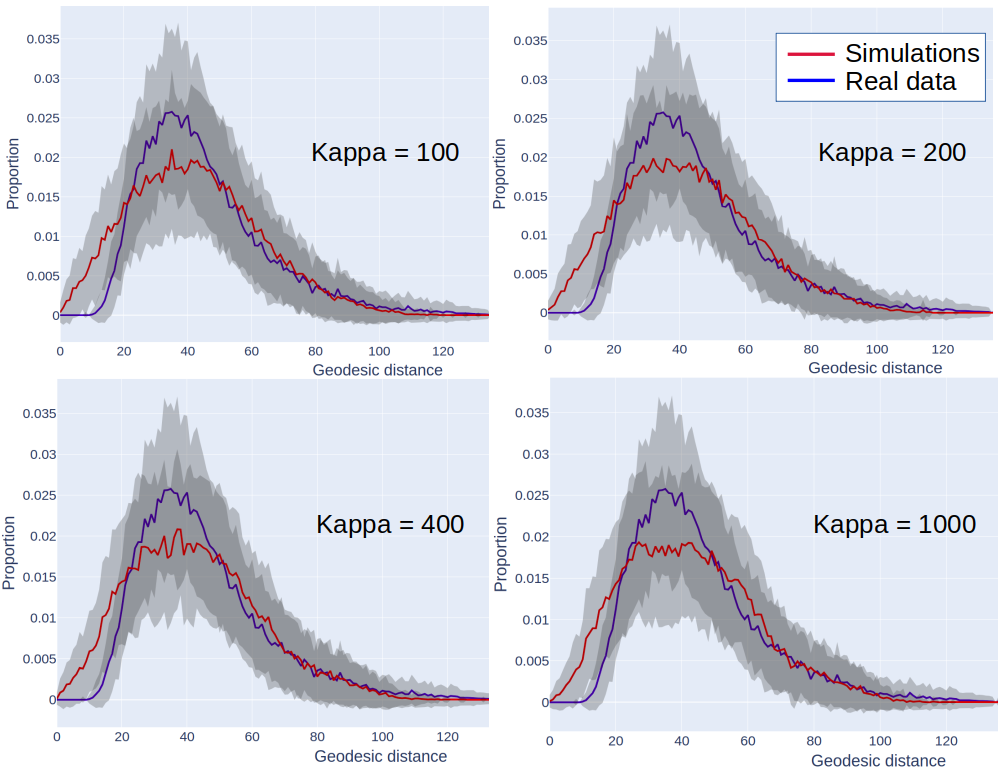

Supplement: S4 Fig — Distribution for geodesic distance in the simulated and real population of 137 graphs. The shaded region corresponds to standard deviations across graphs in the population. (PNG) [file pone.0293886.s004.png]

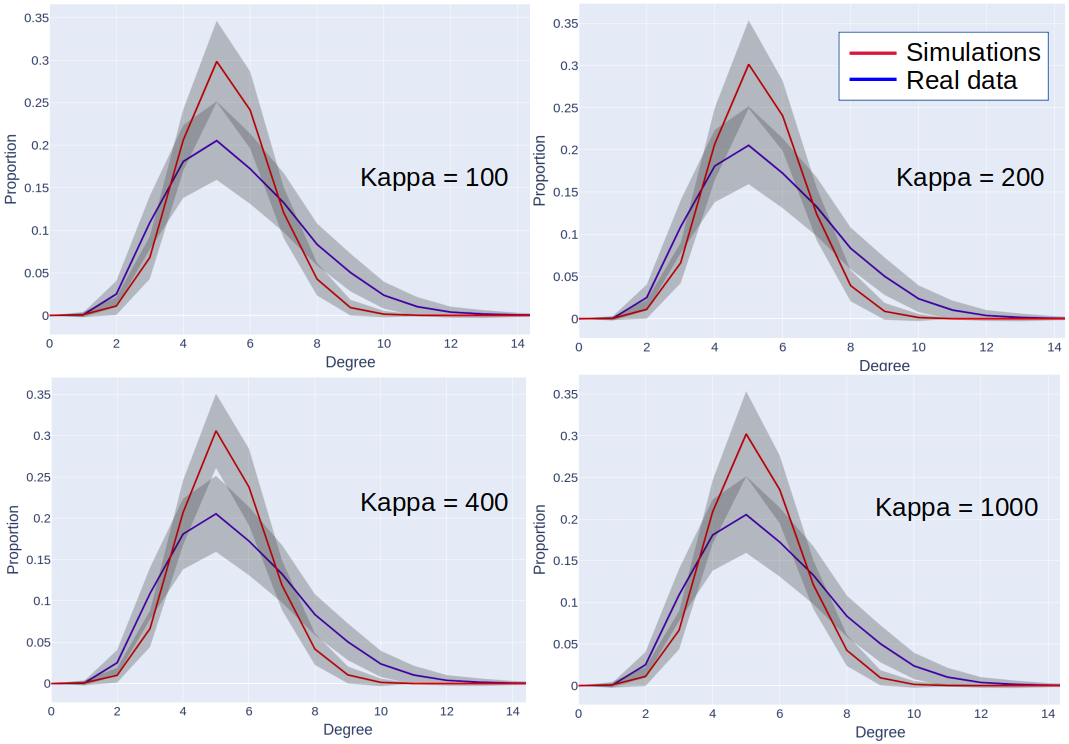

Supplement: S5 Fig — Degree distribution in the simulated and real population of 137 graphs. The shaded region corresponds to standard deviations across graphs in the population. (PNG) [file pone.0293886.s005.png]
